# Assessing the Effect of Pineapple Extract Alone and in Combination With Vancomycin on *Streptococcus sanguis*

**Published:** 2012-10-07

**Authors:** Hengameh Khosropanah, Abdollah Bazargani, Hooman Ebrahimi, Kimia Eftekhar, Zahra Emami, Samira Esmailzadeh

**Affiliations:** 1 Department of Periodontology, Shiraz University of Medical Sciences, Shiraz, IR Iran; 2 Department of Bacteriology and Virology, Shiraz University of Medical Sciences, Shiraz, IR Iran; 3 Department of Oral Medicine, Shiraz University of Medical Sciences, Shiraz, IR Iran

**Keywords:** Vancomycin, *Streptococcus sanguis*, Periodontitis

## Abstract

**Background:**

Periodontitis is a major problem that affects a large number of patients in the society. Various treatment alternatives have been proposed to control this pathologic condition. *Streptococcus sanguis* is one of the countless pathogens involved in periodontitis.

**Objectives:**

The current study aimed to assess the efficacy of pineapple extract per se and in synergy with vancomycin on the growth activity of *S. Sanguis*.

**Materials and Methods:**

An experimental study was designed to determine the minimum inhibitory concentration (MIC) of Pineapple extract per se and in conjunction with vancomycin. The study was carried out in three stages. Serial concentrations of the aqueous pineapple extract, vancomycin, and pineapple plus vancomycin were prepared by broth microdilution technique respectively and were exposed to the standard laboratory strain of S. sanguis (10556 ATCC). The lowest concentration of the pineapple extract and the mixed pineapple/vancomycin solution which inhibited bacterial growth was recorded as the MIC.

**Results:**

The minimum inhibitory concentration of vancomycin was determined 1 µg/ ml. The pineapple extract failed to show any inhibitory effects per se, however, once added to vancomycin, it reduced the MIC to 0.5µg/ml.

**Conclusion:**

Prescription of pineapple extract along with antibiotics increases the antibacterial effects of the drug, therefore reduces the minimum inhibitory concentration of the antibiotic.

## 1. Background

Gingival and periodontal diseases have long been major health problems affecting human beings throughout history. Epidemiologic studies show that severe/destructive periodontal diseases with substantial bone loss have affected the early generations of mankind in the ancient times (1). Periodontal disease is the tissue response to pathogenic microorganisms in dental plaque and is considered as a chronic condition which starts with gingival inflammation and may gradually develop towards severe soft and hard tissue deterioration and tooth loss (2, 3). Plaque induced gingivitis is the most common form of the disease characterized by the infiltration of immune cells in response to the presence of virulent microorganisms in dental plaque. Medications and local or systemic factors may affect the severity and duration of the inflammatory reaction, thus interfering with the process of plaque removal (4).

*Streptococcus sanguis* is a gram positive anaerobic bacterium which constitutes the normal flora of the oral cavity. However, it has also been identified as one of the causative microbial agents in periodontal diseases (5, 6). Identifying the major cause of periodontitis is a crucial step in guiding the clinicians to take the necessary measures to control microbial load in the affected site. Antibiotics are the most common means of treating periodontitis (6). However, like other chemical agents, they may elicit adverse effects in patients. Hence, clinicians have shown greater tendency toward prescribing herbal remedies to control pathologic conditions and there is countless evidence comparing the efficacy of herbal products against biochemical agents respectively (7).

Pineapple (Ananas comosus) is a tropical plant belonging to the family of Bromeliacea (8). The medical uses of this plant include, but are not limited to anti-inflammatory and proteolytic agent, antihelminthic agent and in some practices it may also be implied to induce abortion. Bromelain is the term which refers to a complex of enzymes derived from A.comosus which is used to treat a variety of pathologic conditions and is considered as a substitute for glucocorticoids, Non-Steroidal Anti-inflammatory agents (NSAIDs) and immunomodulators (9-11). Although it demonstrates very low toxic effects, researchers have recommended limited usage of Bromelain in patients with hemophilia and liver and kidney problems due to anti-coagulatory effects (12). Although several studies have documented the anti-inflammatory effects of the bromelain complex derived from pineapple extract, there is limited information regarding the therapeutic efficacy of this herbal product to treat periodontal diseases. 

## 2. Objectives

The current study aimed to evaluate the antibacterial effects of pineapple extract alone and in combination with vancomycin to inhibit the growth of *Streptococcus sanguis*.

## 3. Materials and Methods

Laboratory standard strain of *Streptococcus sanguis* (10556 ATCC) was cultured in Brain Heart Infusion broth (BHI broth) and was incubated in 5-10% CO2 at 37oC for 48 hours. The solutions were subsequently subjected to spectrophotometry at 530nm to reach the effective bacterial concentration (0.5 MacFarland). To prepare pineapple extract, 20 gr of pineapple was peeled, cut and blended to yield a homogenous extract. The extract was then sieved several times to yield a transparent homogenous liquid. 10 ml of this liquid was then poured into a 50 ml falcon and placed in a freeze drier (-50oC). The vacuum condition in the freeze drier resulted in the formation of small yellow crystals which were stored at -4oC for subsequent use.

The minimum inhibitory concentration (MIC) of the pineapple extract on S. sanguis was determined by broth microdilution technique. For this purpose, 96-hole microplates (12 columns, 8 rows) were selected. The holes in the first column were filled with 200 µl of Trypticase Soy Broth (TSB) and the remaining columns (from 2 to 12) were filled with 100 µl of TSB respectively. The pineapple extract was then mixed with TSB to yield a solution with 16384 µg/ml concentration. 100 µl of this solution was added to the holes C, D and E in the second column. The containments of holes C, D and E were thoroughly mixed using a sampler and 100 µl of the solution was transferred to the succeeding holes; this process was repeated until the11th column resulting in serial dilution of the pineapple extract. Hence, each hole contained twice as much pineapple extract as the succeeding hole. An identical procedure was applied to holes F,G and H to dilute the antibiotic vancomycin. The initial concentration of vancomycin was 8 µg/ml (4 µg/ml tablets, Mast, England). Finally, 100 µl of bacterial suspension with 105 bacteria was added to every hole, rendering 4 µg/ml of antibiotic and 4096 µg/ml of the pineapple extract in the relative holes in the second column respectively. The first column contained only the culture medium and served as negative control to maintain sterility and the last column merely contained bacteria and served as positive control to verify bacterial growth. The plates were subsequently incubated for 48 hours (5-10% CO2, 37oC) and subjected to visual inspection with bare eyes. The last hole, which failed to show any discoloration, was considered as the minimum inhibitory concentration (MIC). In order to minimize potential errors, all stages were repeated three times (in three rows) and in case of any inconsistency, the investigation was repeated. Discoloration in the holes indicated inadequate concentration of the extract or the antibiotic to diminish bacteria.

In order to confirm the antimicrobial effect of pineapple, higher concentrations of the solution were tested in the first two rows of holes following an identical microdilution pattern with a slight difference; i.e. the initial concentration was 8192 µg/ml. To determine the sensitivity of S. sanguis to vancomycin in presence of serial concentration of pineapple extract and to assess the boosting effect of pineapple extract on the antibiotic in vitro, the identical broth microdilution pattern was utilized. The first row served as negative control and was filled only with 200 µl of the TSB culture media. All remaining holes were subsequently filled with the pineapple extract in microdilution pattern starting at a concentration of 16384 µg/ml. Then, vancomycin solution with concentration of 4 µg/ml was added to the first two rows to achieve 1µg/ml (MIC) concentration of antibiotic. In the next 2 rows vancomycin solution was added to achieve 0.5 µg/ml (sub MIC) concentration and the next 2 rows were prepared by the same method with the final vancomycin concentration of 0.25 µg/ml and in the 4th 2 rows, final concentration of vancomycin was 0.125 µg/ml. Following the same instruction, in another plate, serial concentration of pineapple extract was combined with vancomycin final concentration of 0.06 µg/ml, in the 1st two rows, and vancomycin final concentration was 0.03 µg/ml in the 2nd 2 rows. Each row was designed to have identical concentrations of vancomycin with different concentrations of pineapple extract to eliminate the confounding effect of vancomycin and merely assess the effect of different concentrations of pineapple extract. The plates were incubated for 48 hours (5-10% CO2, 37oC) and then evaluated with bare eyes. Different concentrations of vancomycin were also evaluated in a different plate to determine the minimum inhibitory concentration of the antibiotic on S. sanguis. The last hole which failed to show any evidence of bacterial growth, was considered as the minimum inhibitory concentration against bacterial growth. 

## 4. Results

According to the results of the first part of the experiment, the minimum inhibitory concentration of vancomycin was determined 1 µg/ml. None of the holes containing different concentrations of pineapple extract ranging between 16384µg/ml and 4096 µg/ml were deemed effective in bacterial growth inhibition. The second stage of the experiment, studying serial concentrations of pineapple extract up to 8192 µg/ml, also failed to show any success in bacterial growth inhibition. The researchers failed to dilute further concentrations of pineapple extract in trypticase soy broth. The third plate presented the effect of different concentrations of pineapple extract combined with vancomycin on bacterial growth. It was observed that MIC of vancomycin decreased from 1 µg/ml to 0.5 µg/ml when combined with pineapple extract at concentrations up to 32 µg/ml. However, lower concentrations of vancomycin (0.25, 0.12, 0.06, 0.03 µg/ml) failed to demonstrate any effective antibacterial activities when combined with pineapple extract.

## 5. Discussion

Herbal products are employed in industrial and medicinal applications due to their limited adverse effects, researchers have increasingly turned toward studying different aspects of pharmaceutical usages of these products (13). The current study aimed to assess the effect of pineapple extract alone and in combination with vancomycin on the standard laboratory strain of *Streptococcus sanguis* (ATC 10556) as the initial colonizer in dental plaque. This microorganism is shown to enter blood stream and colonize in arteries and valves and is known as one of the major causes of sub-acute bacterial endocarditis (14). The current study demonstrated that although pineapple extract containing bromelain enzyme complex is not effective in inhibiting bacterial growth by itself, once combined with vancomycin, it is able to reduce the minimum inhibitory concentration (MIC) of vancomycin to half its MIC when prescribed alone. Bromelain is a proteolytic enzyme complex which has long been used in Central and South America to enhance wound healing, and inflammation reduction. It is derived from pineapple stem and extract. The German Commission E approved the anti-inflammatory effects of bromelain in treating swelling and inflammation after surgeries (15). Bromelain is also believed to increase the absorption of antibiotics which leads to better distribution of the drug in tissues and thus decreases the potential adverse effects associated with antibiotic toxicity (11). Scientists claim that bromelain may also exhibit antibacterial properties in addition to anti-inflammatory effects (16).

Currently the most common means of treating periodontal diseases is mechanical removal of infected tissues and the causative microorganisms. However, certain cases of periodontitis may require additional antimicrobial agents in order to eliminate all the pathogens in the infected site (17). Chlorhexidine, antibiotics, and enzyme complexes are among the most frequently used antimicrobial agents, nevertheless, they are associated with certain disadvantages which limit their long-term application in cases of chronic periodontitis (13). Recent studies have evaluated the sensitivity of different bacteria to herbal and natural agents (18). The present study evaluated the minimum inhibitory concentration of bromelain alone and in combination with vancomycin on bacterial growth by broth microdilution technique. Broth dilution (micro or macro) is a reliable technique used to quantify the efficacy of antimicrobial agents in vitro (19). Due to its simplicity and precision of results, broth microdilution is one of the most common techniques to evaluate MIC (13). The researchers failed to identify any studies on the antibacterial properties of bromelain alone and synergically with vancomycin; however, Wolinsky et al. and Babpour et al. investigated the antibacterial effects of other herbal products i.e. Neem and Thymus vulgaris, M. officinalis, R. coriaira, M. grandiflora on the standard strain of S. sanguis respectively. They failed to observe any significant inhibitory effect in the aqueous extract; however, the alcoholic (ethanolic and methanolic) extract of the last three plants displayed significant inhibitory effects on the growth of the microorganism (20, 21). Another study evaluated the synergistic effect of an ethanol extract of T. ulmifolia and aminoglycosides on methicillin resistant strain of S. aureus and demonstrated that the addition of 32 µg/ml of the ethanol extract dramatically reduced the MIC for gentamicin and kanamycin (7). Alcohol has traditionally been used as a solvent to enhance the release of useful elements from plants fibrocellulose. Yet many people disfavor alcoholic herbal extracts because of its bad taste, adverse hepatic effects and other associated complications such as xersotomia, burning mouth syndrome, increased risk of oral cancer and neuropathic effects (22). The majority of herbal extracts in the market contain 30-70% of alcohol; this amount of alcohol may alter the antibacterial effects of the extract and thus, many favors toward the use of aqueous herbal extracts (22). Al-hebshi et al. reported that the addition of 5 µg/ml of the aqueous crude extract of C. edulis (Khat) significantly reduced the MIC of tetracycline to half its required amount to inhibit the growth of S.sanguis (18). However, such extracts have shown to increase the risk of oral cancer in excessive usage (23). Since pineapple extract may have acidic pH, it was better if the effect of pH on the growth of S. sanguis had been estimated. So it is recommend that other studies consider the point.

The current study demonstrated that prescription of pineapple extract along with antibiotics increases the antibacterial effects of the drug and thus reduces the minimum inhibitory concentration of the antibiotic. Hence, lower doses of the antibiotic would be needed to treat the streptococcal infection. 
